# The journey of boswellic acids from synthesis to pharmacological activities

**DOI:** 10.1007/s00210-023-02725-w

**Published:** 2023-09-23

**Authors:** Ehab A. Ragab, Mohammed F. Abd El-Wahab, Ahmed S. Doghish, Rania M. Salama, Nermin Eissa, Samar F. Darwish

**Affiliations:** 1 Department of Pharmacognosy and Medicinal Plants, Faculty of Pharmacy, Al-Azhar University, Cairo, 11884 Egypt; 2 Department of Biochemistry, Faculty of Pharmacy, Badr University in Cairo (BUC), Badr City, 11829 Cairo Egypt; 3 Biochemistry and Molecular Biology Department, Faculty of Pharmacy (Boys), Al-Azhar University, Nasr City, 11231 Cairo Egypt; 4 Pharmacology and Toxicology Department, Faculty of Pharmacy, Misr International University (MIU), Cairo, Egypt; 5 Department of Biomedical Sciences, College of Health Sciences, Abu Dhabi University, P.O. Box 59911, Abu Dhabi, United Arab Emirates; 6 Pharmacology & Toxicology Department, Badr University in Cairo (BUC), Badr City, 11829 Cairo Egypt

**Keywords:** Boswellic acid (BA), Pharmacokinetics, Molecular targets, Pharmacotherapeutic actions

## Abstract

There has been a lot of interest in using naturally occurring substances to treat a wide variety of chronic disorders in recent years. From the gum resin of *Boswellia serrata* and *Boswellia carteri*, the pentacyclic triterpene molecules known as boswellic acid (BA) are extracted. We aimed to provide a detailed overview of the origins, chemistry, synthetic derivatives, pharmacokinetic, and biological activity of numerous *Boswellia* species and their derivatives. The literature searched for reports of *B. serrata* and isolated BAs having anti-cancer, anti-microbial, anti-inflammatory, anti-arthritic, hypolipidemic, immunomodulatory, anti-diabetic, hepatoprotective, anti-asthmatic, and clastogenic activities. Our results revealed that the cytotoxic and anticancer effects of *B. serrata* refer to its triterpenoid component, including BAs. Three-O-acetyl-11-keto—BA was the most promising cytotoxic molecule among tested substances. Activation of caspases, upregulation of Bax expression, downregulation of nuclear factor-kappa B (NF-kB), and stimulation of poly (ADP)-ribose polymerase (PARP) cleavage are the primary mechanisms responsible for cytotoxic and antitumor effects. Evidence suggests that BAs have shown promise in combating a wide range of debilitating disease conditions, including cancer, hepatic, inflammatory, and neurological disorders.

**Graphical abstract:**

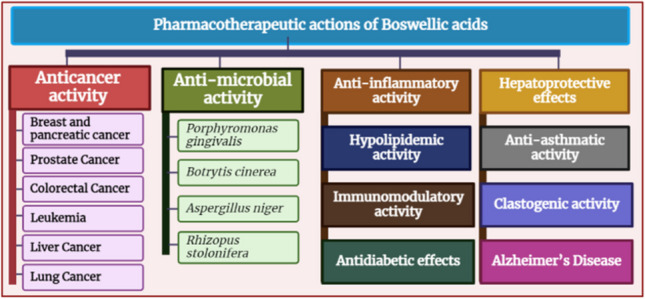

## Introduction

Boswellic acid (BAs) are important medicinal and therapeutic agent obtained from frankincense or olibanum since ancient times. BAs are related chemically to pentacyclic triterpenoids and belong to the ursane and Olean groups. In Arabian and western cultures, frankincense is burned on charcoal, and incense smoke is emitted with a characteristic smell (Duke 2008). Frankincense is an oleogum resin that exudates from the trunk of the trees of various *Boswellia* species (Addisalem et al. 2016). BAs are present in almost all *Boswellia* species (Duke 2008, Al-Harrasi et al. 2021) and are considered chemotaxonomic markers for the genus *Boswellia*.

Various species of *Boswellia* are employed in traditional medicine for the treatment of many diseases such as inflammation, arthritis, joint pain, muscle pain, gout, chronic pain syndrome, chronic bowel diseases, stomach aches, colds, cough, asthma, bronchitis, fever, cancer, and cerebral edema as well as it is used against dental infections and as a tonic for the digestive system (Abercrombie 1985, Wahab et al. 1987, Wichtl 2004, Ammon 2006, Takahashi et al. 2012, Hamidpour et al. 2015, Addisalem et al. 2016). The anti-inflammatory properties of the plant belonging to genus *Boswellia* are related to its contents of BA derivatives in particular acetyl-11-keto-β-BA (AKBA) and 11-keto-β-BA (KBA) (Safayhi et al. 1992, Pawar et al. 2011, Du et al. 2015).

In vitro studies including antioxidant activity (Hartmann et al. 2012), anti-inflammatory, anti-edema, antinociceptive and analgesic (Fan et al. 2005, Mothana 2011), antiarthritic (Sumantran et al. 2011, Ammon 2016), antibacterial (Abdallah 2009, Raja et al. 2011), antiviral (von Rhein et al. 2016), antithrombotic (Kokkiripati et al. 2011), antitrypanosomal (Atawodi et al. 2011), anticancer (Winking 2008), antidiarrheal (Borrelli et al. 2006), antiulcer (Zeeyauddin et al. 2011), antidiabetic (Azemi et al. 2012), anti-hyperlipidemic (Liu et al. 2013), antidepressant, anti-anxiety (Liu et al. 2013), neuroprotective (Ding et al. 2014), and hepato-protective (Jyothi et al. 2006) activities of *Boswellia* extracts have been reported.

Additionally, some *Boswellia* extracts are used for the treatment of collagenous colitis (Madisch et al. 2007), ulcerative colitis (Gupta et al. 1997, Peng et al. 2022), chronic colitis (Gupta et al. 2001), Crohn’s disease (Alam et al. 2012, Hartmann et al. 2014), chronic cluster headache (Lampl et al. 2012), hepatitis (Safayhi et al. 1991), asthma (Poeckel and Werz 2006), pulmonary fibrosis (Ali and Mansour 2011), skin infections, psoriasis, eczema (Togni et al. 2014), and for skin whitening and in reducing wrinkle (Al-Harrasi et al. 2014), intelligence improvement, and enhancement of memory and immune response (Farshchi et al. 2010, Gupta et al. 2011).

When compared to the parent BAs, the pyrolysate products of frankincense resin demonstrated less anticancer potential against MDA-MB-231 breast cancer (BC) cells, demonstrating that the antiproliferative activity is inversely related to loss of functional groups (Al-Harrasi et al. 2014). Many reviews were reported previously concerning the chemistry and biological activities of the oleogum resin obtained from different species of *Boswellia* (Poeckel and Werz 2006, Sharma et al. 2007).

This review gives a more comprehensive study of BA and its derivatives including the sources, chemistry, synthetic derivatives, pyrolysate products pharmacokinetic, and biological activities of various *Boswellia* species and their BA and its derivatives.

## Pharmacognostical characteristics of BAs

*Boswellia* is a genus in the family Burseraceae that contains around 25 different species found in tropical regions, Africa, and India (Hedberg and Edwards 1989, Siddiqui 2011). *Boswellia serrata* is one of the common species, commonly known as Indian olibanum, kundur, loban, or salaiguggal was subjected to intensive phytochemical and biological investigation. Other species belonging to the genus *Boswellia* include *B. cateri*, *B. carterii*, *B. sacra*, *B. frereana*, *B. bhau-dajiana*, *B. rivae*, *B. papyrifera*, *B. neglecta*, *B. odorata*, *B. ovalifoliolata*, and *B. dalzielli* which were to some extent investigated chemically and biologically (Siddiqui 2011). Different locations give different common names to *Boswellia*; in India, it is called “salaigugal” and in Arabic, it is known as “luban”. Different types of species give also different common names; in Somalia; frankincense from *Boswellia frereana* is called “jagcaar,” while frankincense from *Boswellia papyrifera* is known as “boido” and that from *Boswelli acarterii* is known as “moxor” (Hedberg and Edwards 1989).

Frankincense is an oleogum resin that exudes from the trunk of the large branched trees of various *Boswellia* species after peeling or after a series of incisions. This oleogum resin exudes as a milky substance that solidifies in exposure to air to give amorphous yellowish-white lumps or tears with an aromatic odor and bitter taste (Senghani and Patel 2013, Addisalem et al. 2016). The physical characteristics of the solidified resin as the color, hardness, and texture vary from one species to another and within different grades of the same species. Furthermore, the yield and chemical composition of the oleo gum resin vary according to the habitat, and time of collection (Vuddanda et al. 2016).

## Oleo-gum resin of *Boswellia* species

The chemical composition of the oleo-gum resin obtained from different *Boswellia* species is about 30–60% resin, 5–10 volatile oil, and 25–30% gum. The volatile oil is a mixture of terpenoid substances mainly mono-, di-, and sesquiterpenes, as well as phenolic compounds. Serratol is a diterpene alcohol present in the volatile oil components of the oleo-gum resins which are responsible for its odor. Gum is composed of disaccharides, oligosaccharides, and polysaccharides in which arabinose, xylose, and galactose are the major monosaccharide units. The chief constituents of resin are monoterpenes, diterpenes, tetracyclic triterpenes, and pentacyclic triterpenes. Among the pentacyclic triterpenes, the more medicinal active compounds are the BA and its derivatives (El Khadem et al. 1972, Al-Harrasi and Al-Saidi 2008, Hamidpour et al. 2013, Senghani and Patel 2013, Addisalem et al. 2016, Vuddanda et al. 2016).

Several chromatographic techniques such as GC/MS, headspace solid-phase micro-extraction-GC/MS (SPME-GC/MS), reversed-phase HPLC, and HTPLC have been used for analyses of oleogum resin constituents, separation, and identification of individual BAs (Krohn et al. 2001, Büchele et al. 2003, Hamm et al. 2003, Mathe et al. 2004, Mathe et al. 2007, Shah et al. 2007a).

## Chemistry of BAs

**Fig. 1:**
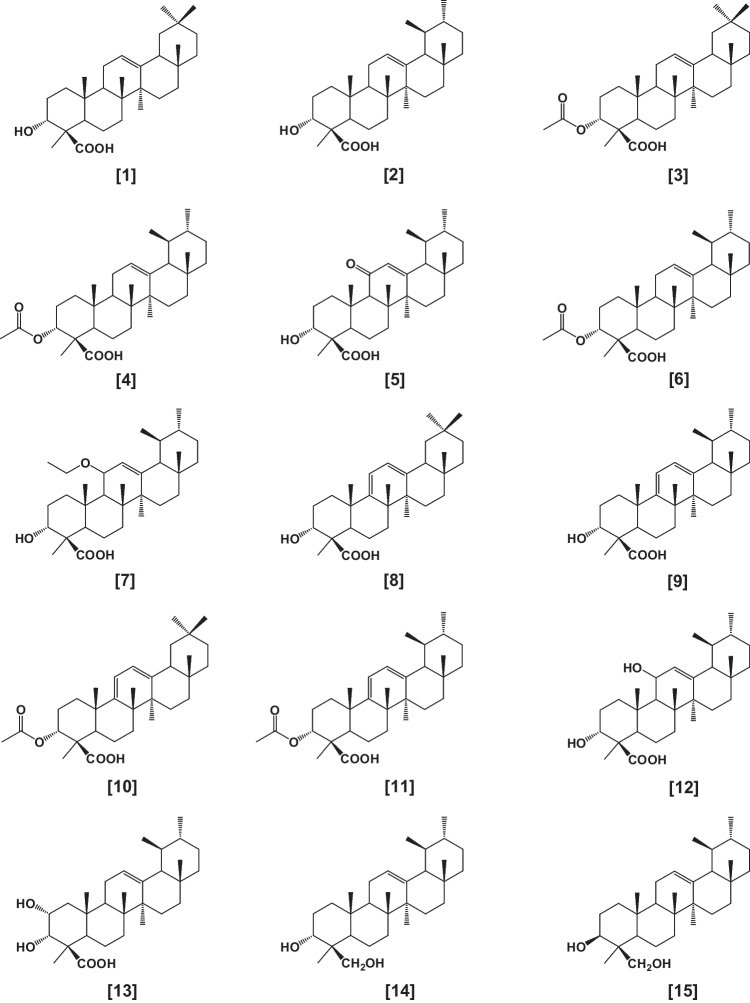
Derivatives of BAs

The resin part of oleogum resin of various *Boswellia* species contains many derivatives of pentacyclic triterpenic acids, known collectively as BAs which are related chemically to 3-hydroxyolean-12-ene-23-oic acid (α-BA) and 3-hydroxyurs-12-ene-23-oic acid (β-BA). BAs are considered the most effective principle responsible for the medicinal activity of frankincense. The main BAs and their derivatives which have been isolated from various *Boswellia* species (particularly *B*. *serrata*, *B*. *carteri*, and *B*. *sacra*) included α-BA [**1**], β-BA [**2**], acetyl-α-BA (Aα-BA) [**3**], acetyl-β-BA (Aβ-BA) [**4**], 11-keto-β-BA (KBA) [**5**], AKBA [**6**], as well as and 11α-ethoxy-β-BA [**7**]. In addition to diene derivatives, namely 9,11-dehydro-α-BA [**8**], 9,11-dehydro-β-BA [**9**], acetyl-9,11-dehydro-α-BA [**10**], acetyl-9,11-dehydro-β-BA [**11**] which is believed to originate from their corresponding 11-hydroxyBA [**12**] (Fig. 1) (Ammon et al. 1991, Schweizer et al. 2000, Büchele et al. 2003, Al-Harrasi et al. 2013).

Further BA derivatives have been isolated from acidic and neutral fractions of the gum extract and identified as 2,3-dihydroxy-urs-12-ene-24-oic acid [**13**] and urs-12-ene-3-α,24-diol [**14**], and its isomer urs-12-ene-3-β,24-diol [**15**] (Mahajan et al. 1995).

Some physical, chemical, and spectral properties of the main BAs and their derivatives present in high amounts in different *Boswellia* species have been reported (Vuddanda et al. 2016, Iram et al. 2017). The configuration of C-3 hydroxyl and C-24 carboxyl groups in BA was determined to be axially oriented (Allan 1968). The presence of the 11-keto group in BA is essential to fit with the receptors to induce anti-inflammatory activity, while replacement of the 11-keto group by a methylene group or reduction of this group to alcohol or deacetylation of the 3-acetyl-11-keto derivatives will decrease the activity. BA with a reduced 11-keto group became more effective in the induction of apoptosis and inhibition of topoisomerase enzymes (Sailer et al. 1996, Glaser et al. 1999, Hussain et al. 2017).

## Synthetic derivatives of BAs

**Fig. 2:**
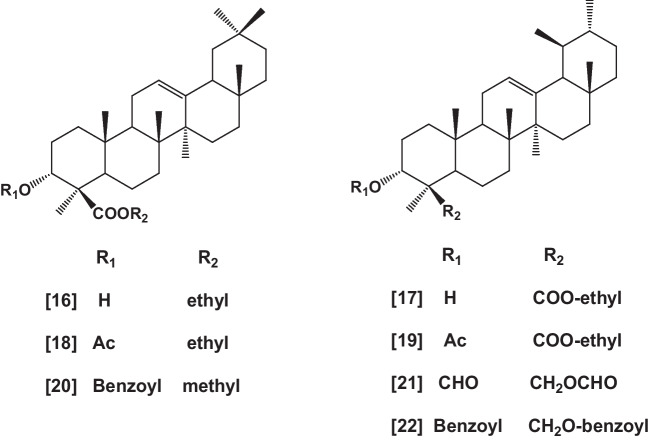
Synthetic derivatives of BAs

Several synthetic derivatives of α- and β-BAs such as the ethyl ester of α- and β-BAs, the ethyl ester of acetyl α- and β-BAs, and the methyl ester of benzoyl α-BA, in addition to the diformate and the dibenzoate of β-BA are prepared (Fig. 2), e.g., ethyl α-BA [**16**], ethyl β-BA [**17**], ethyl acetyl α-BA [**18**], ethyl acetyl β-BA [**19**], methyl benzoyl α-BA [**20**], diformyl β-BA [**21**], and dibenzoyl β-BA [**22**] (El Khadem et al. 1972).

**Fig. 3:**
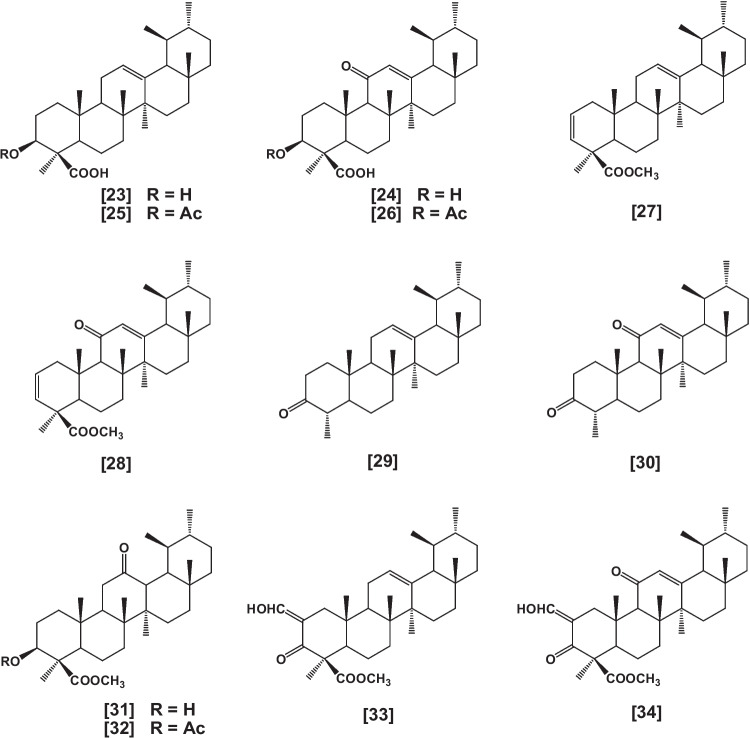
Continue synthetic derivatives of BAs

Other semi-synthetic compounds structurally related to BAs were prepared, and their biological activity has been investigated. Such these compounds are 3-epi analogs of BAs; 3-epi-β-BA [**23**] and 3-epi-11-keto-β-BA [**24**] and their corresponding acetates; 3-epi-acetyl-β-BA [**25**] and 3-epi-acetyl-11-keto-β-BA [**26**] (Shah et al. 2007a), as well as the dien analogs; methyl urs-2,12-dien-24-oate [**27**], and methyl 11-keto-urs-2,12-dien-24-oate [**28**] (Sarett 1948, Sarett 1949), nor analogs; nor-β-boswellenone [**29**] and nor-β-boswellendione [**30**] (Hairfield et al. 1989), 12-keto analogs; methyl-3α-hydroxy-12-oxo-urs-24-oate [**31**], and methyl-3α-acetoxy-12-oxo-urs-24-oate [**32**] (Budziarek et al. 1951, Hairfield et al. 1989), 2-hydroxy methylene analogs [**33** and **34**] (Xenos and Catsoulacos 1985) (Fig. 3).

**Fig. 4:**
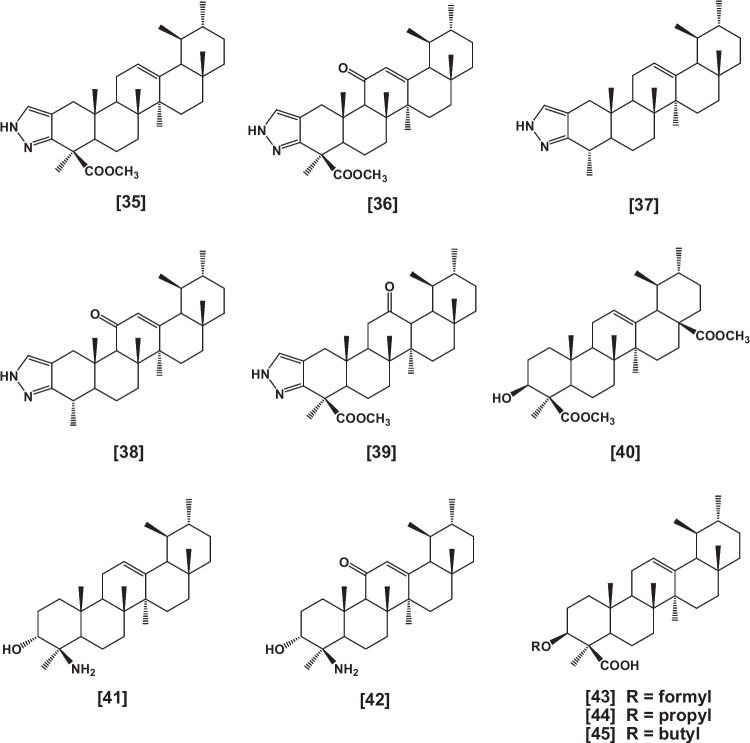
Continue synthetic derivatives of BAs

Heterocyclic pyrazole analogs [**35**–**39**] (Clinton et al. 1962, Shah et al. 2009), analogs having a carboxyl group at C-17 [**40**] (Bore et al. 2000), 4-amino analogs [**41** and **42**] (Shah et al. 2007b), as well as 3-formyl [**43**], 3-propyl [**44**], 3-butyl [**45**] derivatives and their epimers (Shah et al. 2007a, Kumar et al. 2012) were also reported (Fig. 4).

## Pyrolyzed products of BAs

**Fig. 5:**
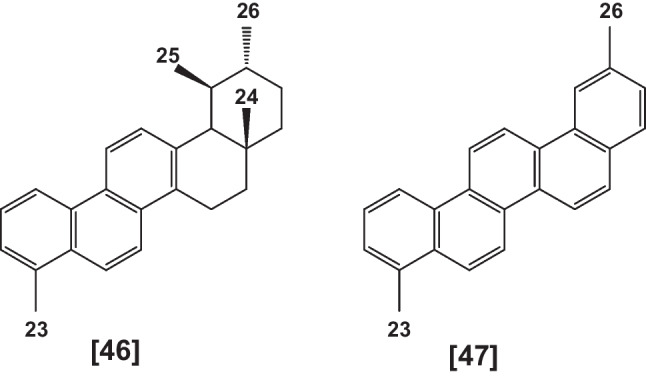
Pyrolyzed products of BAs

Pyrolysis of the resin obtained from *Boswellia sacra* leads to aromatization of ring A caused by dehydration of the C-3 hydroxyl group of BAs, followed by dehydrogenation and demethylation to give tri-aromatic; 1,2,4a,9-tetramethyl-1,2,3,4,4a,5,6,14b-octahydropicene [**46**] and finally penta-aromatic; 2,9-dimethylpicene [**47**] derivatives (Fig. 5). Generally, during pyrolysis of resin hallucinogenic and/or carcinogenic compounds, especially poly-aromatic hydrocarbons are produced (Al-Harrasi et al. 2014). Pyrolysis of *B. serrata*, *B. frereana*, *B. rivae*, *B. neglecta*, and *B. carterii*, using hot charcoal and the GC-MS detection of some volatiles of *B. carterii* was studied (Al-Harrasi et al. 2014, Hussain et al. 2016)*.*

## Pharmacokinetics of BAS

In general, the therapeutic effect of any drug and the determination of its dosage form depends on its bioavailability which in turn depends on its absorption. In this aspect, the low absorption of BA and its derivatives, owing to their lipophilicity especially KBA and AKBA, and extensive metabolism, limited their systemic bioavailability and led to weak therapeutic effects (Sharma et al. 2004, Reising et al. 2005, Alam et al. 2021). Non-acetylated BA derivatives undergo extensive phase I metabolism in the liver, giving hydroxylated derivatives, while acetylated BA derivatives are resistant. KBA and AKBA are poorly absorbed after oral administration; however, AKBA is metabolically stable and is more highly distributed in brain cells than KBA which undergoes extensive phase I metabolism and is highly distributed in plasma (Krüger et al. 2008, Shah et al. 2009, Gerbeth et al. 2013).

Some studies reported that to achieve maximum plasma levels of BAs, they should be administered every 6 h along with fatty meals (Sharma et al. 2004, Skarke et al. 2012). Food intake may affect the absorption, bioavailability, and pharmacokinetics of BAs which in turn affect their medicinal and therapeutic effects (Sharma et al. 2004).

The limited bioavailability of BA and its derivatives can be improved by administering them in the form of nano-emulsion, or with anionic drugs and with standardized meals (Du et al. 2015, Ding et al. 2016, Tambe et al. 2019). The skin permeability to a nano-formula of AKBA is increased in contrast to a normal AKBA gel formula. Formulation of standardized gum resin extract from *Boswellia* with soy lecithin (Casperome^TM^) revealed higher concentrations of BA derivatives in plasma, brain, and various tissues (Hüsch et al. 2013). The absorption of BA complex with phosphatidyl choline is significantly enhanced in comparison with BA due to its amphiphilic nature (Sharma et al. 2010).

Nanoparticles (150–190 nm) of BA were found to be effective and safe in the treatment of prostate cancer (Nandan et al. 2013). Many studies revealed that vinegar-processed frankincense shows higher bioavailability and higher therapeutic effects than raw frankincense (Pan et al. 2015), due to an increased absorption as the vinegar processing of frankincense can cause changes in its physical characteristics, in addition to the acidic character and the heating procedure of vinegar processing increasing the dissolution of alkaline composition in water decoction and cause structural changes in chemical composition (Yin et al. 2017). Recently, the effects of raw frankincense and vinegar-processed frankincense in treating ulcerative colitis were compared which results in the vinegar processing of frankincense improving absorption of its BAs contents as well as its activity through regulating bile acid metabolism mechanism (Peng et al. 2022).

## Pharmacotherapeutic actions of BAs

### Anticancer activity

**Fig. 6:**
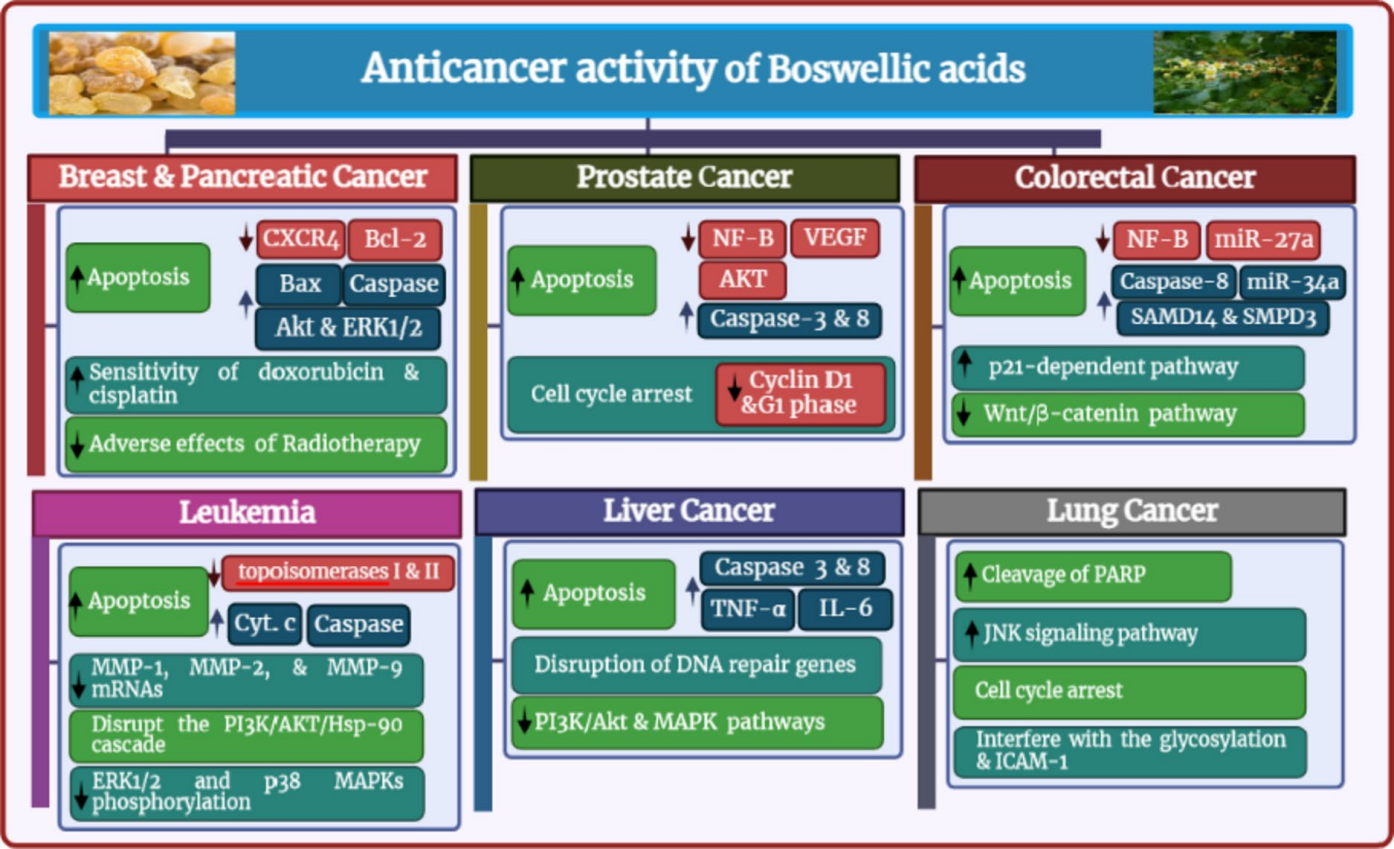
Anticancer activity of BAs

With a very high incidence and fatality rate, cancer is one of the most lethal diseases that affect humans (Al Serwi et al. 2020). However, the majority of currently available medications have serious side effects and are frequently unsuccessful as a result of the emergence of chemo-resistance (Kunnumakkara et al. 2019, Halcrow et al. 2021). This has forced the focus to move toward natural compounds, which have demonstrated promising efficiency against a variety of cancers (Shanmugam et al. 2017). The cytotoxic action of BA against cancer cells has been demonstrated in numerous studies in vitro and in vivo, proving its effectiveness in the prevention and treatment of a variety of cancers (Roy et al. 2016). The modulation of reactive oxygen species (ROS) formation and the resulting endoplasmic reticulum stress is central to BA’s molecular and cellular anticancer activities since it modifies transcription, epigenetics factors, and signal transduction. Cell cycle arrest, growth inhibition, apoptosis induction, and control of inflammation are all the effects of BA’s altered gene expression (Efferth and Oesch 2022). Anticancer activity is seen in Fig. 6.

### Breast and pancreatic cancer

BA demonstrated tumor suppressor ability in BC cells with treatment resistance and metastasis (Suhail et al. 2011). The action of BA in breast tumors was discovered to eliminate the cellular expansion of the tumor, which was associated with a decrease in the level of the protein CXCR4 (Park et al. 2011). When BA triggered apoptosis, it resulted in a markedly reduced level of the anti-apoptotic protein B-cell leukemia/lymphoma 2 protein (Bcl-2) and elevated expression of the pro-apoptotic protein Bcl-2–associated X (Bax). In triple negative breast cancer cells, BA has additional synergistic effects, increasing both the sensitivity and cytotoxicity of doxorubicin and cisplatin (Thummuri et al. 2014). Additionally, BA was well tolerated and helpful in lowering erythema and skin adverse effects in PC patients treated with radiation (Bonucci et al. 2016).

BA decreases viability and induces apoptosis by activating the caspase-dependent pathway in human pancreatic cancer (PC) cell lines, making it a promising therapeutic therapy for pancreatic adenocarcinoma (Ni et al. 2012). In human breast and PC cell lines, BA might inhibit the activation of Ak strain transforming (Akt) and extracellular signal–regulated kinase (ERK)1/2, which both have been suggested as potential molecular targets for treating cancer and improving the response to chemotherapeutic treatments (Becer et al. 2021).

### Prostate cancer

**Fig. 7:**
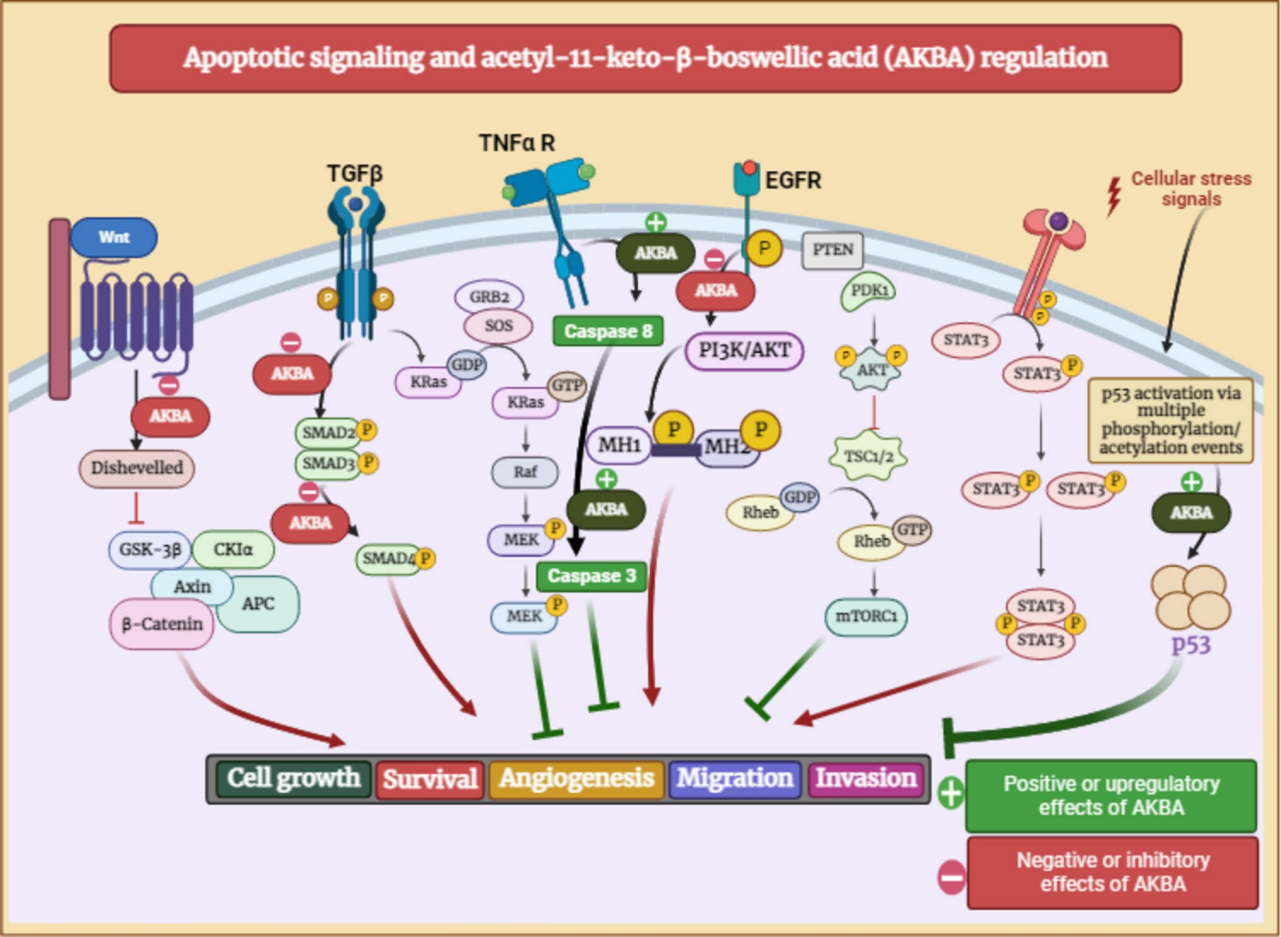
Apoptotic signaling scheme and modulation by acetyl-11-keto-β-boswellic acid (AKBA)

On the in vitro and in vivo basis, AKBA reduced proliferation and induced cell death in androgen-independent prostate cancer cells that were resistant to chemotherapy. BA can reduce the production of NF-B-dependent anti-apoptotic proteins such as Bcl-2 and cyclin D1 by suppressing the constitutively active NF-kB pathway (Syrovets et al. 2005b). Caspase-3 activation was another way that AKBA induced apoptosis in chemotherapy-resistant human PC-3 prostate cancer cells. Meanwhile, in prostate cancer cells (LNCaP), AKBA induces apoptosis via the death receptor 5–mediated pathway. Activation of caspase-3 and caspase-8 as well as the initiation of cleavage of PARP were prompted by the treatment with AKBA (Lu et al. 2008) (Fig. 7).

Further, AKBA affects the androgen receptor by reducing its expression, which is associated with the arrest of the G1 phase of the cell cycle and a decrease in cyclin D1, which inhibits cellular proliferation (Yuan et al. 2008). In prostate cancer, the downregulation of vascular endothelial growth factor receptor 2–mediated angiogenesis caused by BA, led to the inhibition of all downstream protein kinases such as Akt, extracellular signal–related kinase, focal adhesion kinase, and ribosomal protein S6 kinase (Pang et al. 2009). In vivo results also showed promising effects of AKBA, which prevented the multiplication of PC-3 cells that were xeno-transplanted into a chick chorioallantoic membrane and instead led them to undergo apoptosis (Büchele et al. 2006).

### Colorectal cancer

Numerous in vitro and in vivo investigations proved that BA is beneficial in preventing colon cancer. Research on the anti-proliferative and apoptotic properties of BA revealed that it suppressed cell proliferation by activating a p21-dependent pathway (Liu et al. 2006), and induced apoptosis in HT29 colon cancer cells by activating a caspase-8-dependent pathway (Liu et al. 2002b). The β-catenin signaling molecules, which are essential for cancer cell proliferation, were reduced as a result of BA’s anticancer efficacy against colon cancer cells (Becer et al. 2021). Additionally, pre-incubating BA with the phosphatidylinositol-3 kinase (PI3K) inhibitors, LY294002 or wortmannin, significantly increased apoptosis in HT-29 cells (Li et al. 2012). Furthermore, AKBA was found to be more effective than aspirin in preventing both small intestine and colonic polyps in a study comparing the two drugs’ ability to prevent intestinal adenomatous polyposis in mice. Adenomatous polyps were exposed to AKBA, which caused apoptosis and altered the Wnt/β-catenin and NF-κB/ cyclooxygenase-2 (COX-2) pathways (Wang et al. 2014).

Additionally, it was discovered that AKBA significantly reduced Ki-67 and CD31 levels in orthotopically implanted tumors in nude mice, which are markers for colorectal tumor proliferation and differentiation, leading to prevented distant metastasis to other organs such as the liver, lungs, and spleen (Yadav et al. 2012). The investigation on RKO, SW48, and SW480 colorectal cancer cells revealed that AKBA can be a promising new regulator in the prevention and treatment of colorectal cancer. AKBA treatment resulted in a modest genome-wide demethylation, which then allowed simultaneous reactivation of the relevant tumor suppressor genes, including suppressor of mothers against decapentaplegic (Smad)14 and Smad3 (Shen et al. 2012) (Fig. 7). In addition to controlling cell regulatory processes by these genes in colorectal cancer cells, AKBA can also regulate specific cancer-related miRNAs.

miRNAs regulate both protein and gene expression. MiRNAs diminish mRNA stability, including genes involved in cancer processes such as angiogenesis, cell cycle regulation, stress response, differentiation, inflammation, and apoptosis (Ismail et al. 2019, Elrebehy et al. 2022, Ismail et al. 2022, Ismail et al. 2023).

**Fig. 8:**
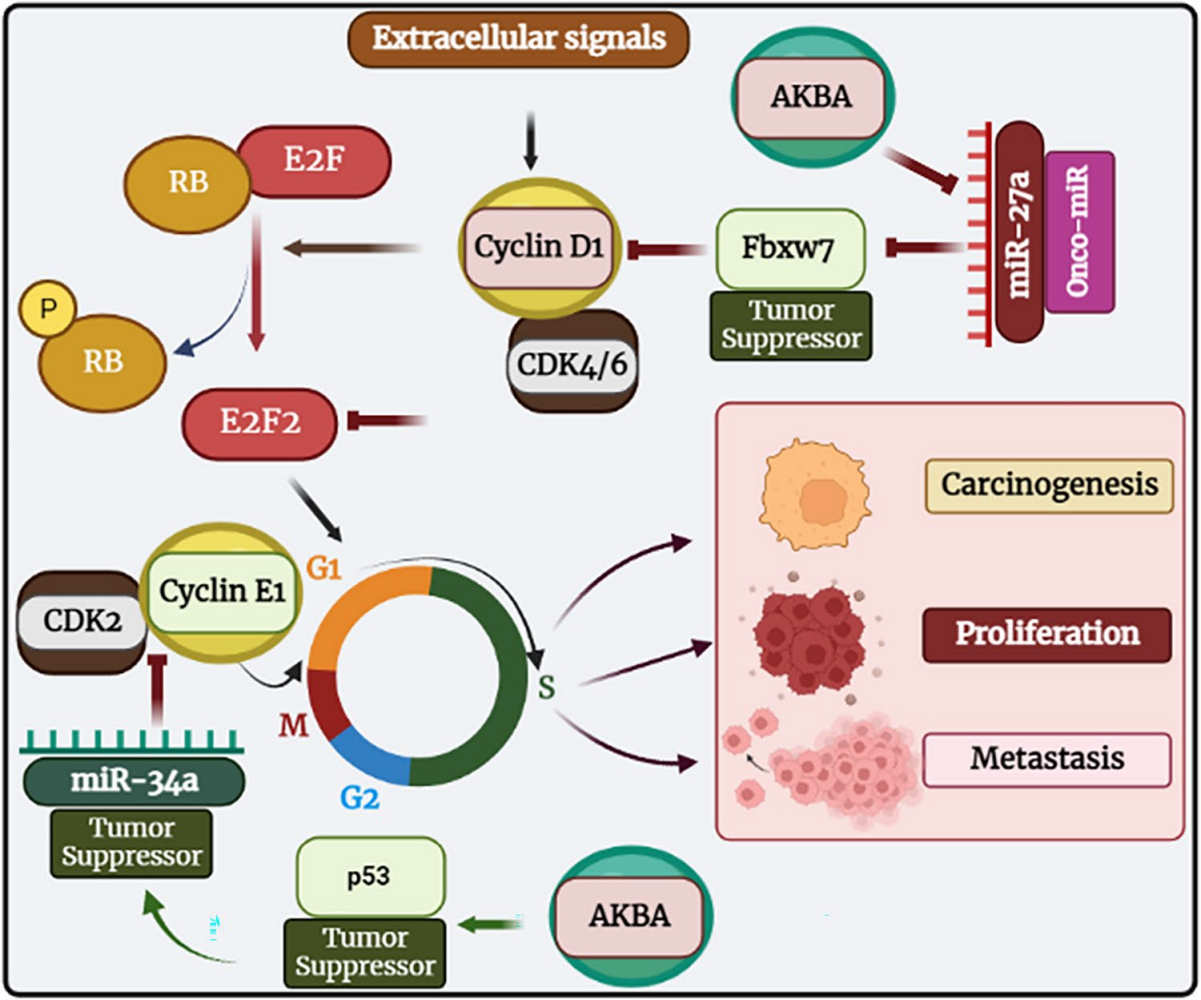
Effects of AKBA on colorectal cancer cells

According to one study, AKBA can increase the expression of tumor-suppressive miRNAs, which in turn can modify the expression of a variety of downstream targets (Takahashi et al. 2012). A xenograft mouse model was recently used in another investigation to confirm the protective activity of AKBA in vivo. The study suggested that AKBA could prevent tumor growth in colorectal cancer cells, which is highly connected with the overexpression of miRNA-34a (tumor suppressor) and the downregulation of miRNA-27a (onco-miRNA) (Toden et al. 2015, Salama et al. 2023) (Fig. 8). In CRC cells and cancer stem cell lines, the F-box protein FBXW7, a tumor suppressor, is a direct target of miR-27a. FBXW7 downregulates components of the NOTCH signaling pathway and stimulates the proteasomal degradation of the transcription factors MYC, cyclin D1, and JUN in CRC stem cells. As a result, miR-27a inhibits FBXW7, which leads to increased MYC, JUN, and NOTCH signaling, cell proliferation, and suppression of secretory lineage differentiation (Babaei-Jadidi et al. 2011). Tumors in mice treated with either AKBA alone or a combination of curcumin and AKBA showed reduced expression of miR-27a. Also, target genes of this miRNA were evaluated for expression in xenograft tumor tissues. This therapy altered the expression of FBXW7, c-Myc, CDK6, and CyclinE1. According to these results, AKBA’s suppression of miR-27a occurs independently of p53 activation (Toden et al. 2015).

### Leukemia

Different leukemic cell lines, including K562, THP-1, ML-1, U937, SKNO-1, and NB4 cells, were used to examine the anticancer efficacy of BA. The induction of apoptosis by the BA treatment was shown to have cytostatic and cytotoxic effects. To reveal its mechanism, BA was found to attenuate topoisomerases I and II, release cytochrome c, lose mitochondrial membrane potential, activate caspases, and cleave PARP (Chashoo et al. 2011). Additionally, it was noted that the medication lowered the expression of matrix metalloproteinases-1, metalloproteinases-2, and metalloproteinases-9 mRNAs as well as tumor necrosis factor-alpha (TNF-α) and IL-1 secretions decreased the phosphorylation of ERK1/2 and p38 mitogen–activated protein kinase (MAPK) and disrupted the PI3K/AKT/Hsp-90 cascade (Xia et al. 2005, Khan et al. 2012).

### Liver cancer

Multiple cases and fatalities from cancer were attributed to hepatocellular carcinoma (HCC) each year (Vogel et al. 2022). When the effects of BA were examined, it was discovered that in liver cancer cells, they induced apoptosis and inhibited proliferation via the caspase-8-dependent signaling pathway (Liu et al. 2002a, Al Serwi et al. 2020). Additionally, BA increased levels of caspase-3 activity (Fig. 7), TNF-α, and IL-6 when it was given alone or in conjunction with doxorubicin, demonstrating growth-moderating and apoptotic effects in HCC cells (Khan et al. 2014). Caspase-driven apoptosis was shown to be a significant mechanism connected with the anticancer action of these drugs, as an increase in caspase-3 activity correlated extremely well with cytotoxicity in vitro. The cytokines TNF-*α* are also capable of triggering an acute inflammatory response. Through the activation of caspase-3, TNF-*α* may also cause apoptosis and suppress carcinogenesis (Utaisincharoen et al. 2000). Combined with an increase in caspase-3 activity, the research found that HepG2 and Hep3B cells secreted more TNF-*α* when the dosage was raised. These findings confirmed that caspase-3 is involved in TNF-*α* -induced apoptosis of HCC cells (Khan et al. 2014). There is evidence that oxidative stress caused by doxorubicin (anticancer) causes an uptick in IL-6 expression. Although B. serrata has been shown to reduce inflammation in healthy tissue, the research indicates that it may instead promote it in cancer cells (Khan et al. 2014). Additional research into nonapoptotic signaling pathways is required to elucidate the reasons behind the elevated IL-6 level seen with boswellic and combined treatment.

Recent research revealed that a novel mechanism by AKBA contributes to the reduction of the growth in HCC cells by promoting early senescence through DNA damage response coupled with the disruption of DNA repair genes (Wang et al. 2020). Additionally, the anti-HCC actions of frankincense and myrrh were recently found to be mediated through PI3K/Akt and MAPK signaling pathways, emphasizing the possibility of these BAs compounds as a prospective treatment for HCC (Zheng et al. 2022).

### Lung cancer

H446 cells were used in an in vitro investigation to test the anticancer potential of BA derivatives. Researchers found that BA inhibited the growth of lung cancer (LC) cells by stimulating the c-JUN N-terminal kinase (JNK) signaling pathway, leading to the cleavage of PARP (Huang et al. 2018). Furthermore, BA could trigger the cleavage of PARP in HOP-62 LC cells. As a result of the therapy, LC cells underwent activation of apoptosis and cell cycle arrest (Qurishi et al. 2012). According to recent studies, the AKBA may improve membrane fluidity and accompanying lipid content in benzo(a)pyrene-induced lung carcinogenesis (Bhardwaj et al. 2019), as well as increase the sensitivity of non-small cell LC cells to cisplatin by arresting the cell cycle, inducing apoptosis, and suppressing autophagy via p21-dependent signaling pathway (Lv et al. 2021). Additionally, novel research found that AKBA improved the sensitivity of radiation-resistant LC cells via controlling the maspin-mediated AKT/FOXO1/p21 pathway (Gong et al. 2022). It is important to note that in human lung adenocarcinoma A549 cells, β-BA, and AKBA were discovered to interfere with the glycosylation and intracellular trafficking of intercellular adhesion molecule-1 (Nakano et al. 2022).

### Other types of cancer

AKBA has been demonstrated to promote cytotoxicity against meningioma cells produced from primary cell cultures of surgically removed meningioma specimens, and the extracellular-signal-regulated kinases 1 and 2 play a vital role in carcinogenesis (Park et al. 2002, Yong et al. 2002). In another study on U266 cells, frankincense, myrrh, and their bioactive constituents reduce the severity of multiple myeloma via regulating the Janus kinase/signal transducer and activator of transcription (STAT) signaling pathway and metabolome profiling (Gao et al. 2020). Another anticancer mechanism through the phosphatase and tensin homolog/Akt/COX-2 signaling axis, AKBA could prevent the growth of gastric cancer cells and promote apoptotic pathways (Sun et al. 2020). The aforementioned experimental findings suggest that BA is a promising candidate for both cancer therapy and carcinogenesis prevention. However, the conventional medical literature now only has a very small number of publications on BA clinical trials on cancer. Therefore, more clinical studies with a high sample size are required, followed by a careful examination of the results to determine their therapeutic potential.

### Anti-microbial activity

Plants that evolved multi-specific chemical defense mechanisms against pathogen infection from viruses, bacteria, or protozoa proved to have an evolutionary benefit during the history of life on Earth. Due to the numerous adverse effects and microbial resistance of conventional antimicrobial agents, it is crucial to explore and optimize these antimicrobial compounds for therapeutic usage in human patients (Efferth and Oesch 2022). To treat microbial and fungal illnesses, BAs isolated from *B. sacra* and *B. serrata* have historically been employed. *B. sacra* essential oil’s monoterpenoids demonstrated antibacterial efficacy against Propionibacterium acnes, *Pseudomonas aeruginosa*, and *Staphylococcus aureus* in vitro research. Additionally, BA significantly reduced the growth of Malassezia furfur and *Candida albicans* (Di Stefano et al. 2020).

Aflatoxin production by *Aspergillus flavus* and *Aspergillus parasiticus* has been greatly decreased by the inhibitory activity of *B. sacra* extract, resin, and essential oil at a variety of doses. Therefore, the essential oil and resin powder from *B. sacra* can be recommended as safe, natural preservatives to increase the storage life of food goods (El-Nagerabi et al. 2013). A significant antibacterial activity versus *Porphyromonas gingivalis* was discovered in a different investigation using *B. sacra* oleoresin extract (Attallah et al. 2021). Furthermore, the effectiveness of *B. sacra* extracts in conjunction with traditional antibiotics was assessed against a range of bacterial infections that affect the human gastrointestinal system and microorganisms that trigger autoimmune diseases, demonstrating that this combination demonstrated significantly greater efficiency than that of the individual agents alone (Rashan et al. 2021). Recently, *Botrytis cinerea*, *Aspergillus niger*, and *Rhizopus stolonifera*—three fungus species that cause strawberry rot—were successfully eradicated by *B. sacra* essential oil’s promising antifungal properties (Rahmati-Joneidabad and Alizadeh behbahani 2021).

Further, the antimicrobial activity of BA versus 112 isolates of harmful bacteria has been approved. In time-kill, post-antibiotic impact tests, and biofilm susceptibility assays, the most robust antibacterial activity was demonstrated by AKBA, through the breakdown of the microbial membrane structure. These assays revealed a strong activity against Gram-positive pathogens in a concentration-dependent manner (Raja et al. 2011).

### Anti-inflammatory activity

Anti-inflammatory effects of frankincense and its components help treat immunological disorders. The fascinating bioactivities of oleogum resins from *B. carterii* and *B. serrata*, the most studied frankincense species, as well as those from *B. dalzielii* and *B. sacra*, essential oils from *B. dalzielii*’s leaves, and bark extracts from *B. elongata*, demonstrate that the anti-inflammatory benefits are not unique to one particular Boswellia species (Siddiqui 2011).

Reduced oxidative stress was one of the effects seen after treatment with frankincense and its components. Reactive nitrogen species, ROS, and lipid peroxidation have been shown to exist at reduced levels (Ammon 2010). In terms of the immune system, it has been frequently observed that neutrophilic granulocyte invasion, mast cell stabilization, T effector cell differentiation, immune cell infiltration into inflamed cells, and leukocyte-endothelial cell adhesive interactions have all dropped significantly (Sengupta et al. 2011, Beghelli et al. 2017).

Various studies indicated that the BA anti-inflammatory molecular mechanism was through inhibiting inflammatory factors and/or pathways such as prostaglandins (PGs), histamine, leukotriene, and interferon (IFN)-γ (Henkel et al. 2012), in addition to suppressing 5-lipoxygenase (LOX), cytokines, TNF-α, COX-2, and inducible nitric oxide synthetase (iNOS) (Governa et al. 2018, Loeser et al. 2018). Further, BA upregulated free oxygen radicals and boosted antioxidant defense through catalase, glutathione peroxidase, and superoxide dismutase (SOD) (Ammon 2010).

The anti-inflammatory effect of BA is also involving signal transduction and transcription factors inhibition, such as ERK1/2, NF-κB, inhibitory κB kinase (IKK), and MAPK. In addition, BAs could suppress the activity of STAT3, JNK, SMAD2/3/4/7, and IL-1 receptor-associated kinase (Mostafa et al. 2015, Governa et al. 2018, Liu et al. 2018) (Fig. 7).

Taken together, these multiple anti-inflammatory mechanisms led to the successful treatment of several diseases by *Boswellia* extracts and its phytochemicals, such as osteoarthritis, rheumatoid arthritis (Yu et al. 2020), gastric colitis (Gupta et al. 2001), and autoimmune encephalomyelitis (Nadeem et al. 2022), as well as allergic asthma (Liu et al. 2015), non-alcoholic fatty liver disease and renal fibrosis (Zaitone et al. 2015, Liu et al. 2018) (Table 1). These results highlight BA’s potential for treating inflammatory conditions and point to the importance of conducting additional research to establish BA as a natural substitute for synthetic traditional anti-inflammatory medicines.

**Table 1:** Pharmacotherapeutic actions of BAs

Disease	Mechanism	Ref.
Osteoarthritis, rheumatoid arthritis	- ↓ 5-LOX, TNF-α, MAPK/NFκB, matrix metalloproteinase-3	Yu et al. 2020
Colitis	- ↓ 5-LOX, ↑ expression of the absorption-related protein multidrug resistance-associated protein 2, organic anion transporting polypeptide 1B3	Gupta et al. 2001, Madisch et al. 2007 Gupta, et al. 1997, Peng et al. 2022, Gupta et al. 2001
Autoimmune encephalomyelitis	- ↓ NF-κB and iNOS, ↑ Nrf2 and HO-1	Nadeem et al. 2022
Asthma	- ↓ LOX-5, IgE, IL-4, IL-5, IL-13, GATA3/STAT6 axis, enhanced IFN-γ	Siemoneit et al. 2009, Liu et al. 2015, Zhou et al. 2010, Suther et al. 2022, Yugandhar et al. 2018
Renal fibrosis	- ↓ TGF-β1, α-SMA, collagen I and collagen IV, phosphorylated-Smad2/3 (p-Smad2/3) and Smad4	Liu et al. 2018
Hyperlipidemia	- ↓ Cholesterol, LDL, triglyceride, ↑ HDL	Azadmehr et al. 2014, Mehrzadi et al. 2016
Covid-19	- ↑ Lymphocyte count, ↑ oxygen saturation	Barzin Tond et al. 2022
Diabetes	- ↑ Glyburide bioavailability and enhanced its glucose-lowering ability - ↑ Insulin levels and lower serum glucose level - Inhibit granulocyte colony-stimulating factor and GM-CSF and apoptosis of the β cells - Attenuation of MDA and enhancing SOD levels - ↓ IA_2_-A and HbA_1C_ and reduced GAD65	Kherouf et al. 2021 Khan et al. 2022 Schrott et al. 2014 Franić et al. 2020
Hepatotoxicity	- ↓ ALT, AST, LDH, TGF-β, NFκB, TNF-α, IL-6 - ↓ MDA, HO-1, caspase-3, ↑ Nrf2 - ↓ AST, ALT, C-reactive protein, NFκB p65, p-JNK, TLR9 - ↑ PPAR-α/p38 signal, ↓ JNK	Thabet et al. 2022, Ahangarpour et al. 2014 Barakat et al. 2018 Chen et al. 2016, Monir et al. 2022 Xiao et al. 2017
NAFLD	- ↓ ALT, AST, TNF-α, IL-6, COX-2, MDA, 4-HNE, iNOS, ↑ GSH	Zaitone et al. 2015
Psoriasis and eczema	- ↓ NF-kB, IKK	Togni et al. 2014, Wang et al. 2009
Crohn’s disease	- ↓ TNF-α-induced genes, induced-proteolysis - ↓ IL-2, IFN-γ, ↑ IL-4, IL-10 - ↓ TNF-α, IL-1, IL-6, IL-12	Roy et al. 2005 Chevrier et al. 2005 Gayathri et al. 2007, Khajuria et al. 2008a, b
Alzheimer’s disease	- enhance branching of neurites and tubulin polymerization, ↓ axonal degradation - ↓ BACE1 - ↓ hyperphosphorylation and ROS - ↓ 5-LOX and COX enzymes, TNF-α, IL-6	Karima et al. 2010 Wei et al. 2020 Fathi et al. 2016, Bishnoi et al. 2005, Sayed and El Sayed 2016 Marefati et al. 2020

### Hypolipidemic activity

*Boswellia* is a potent hypolipidemic agent, as proven by numerous scientific investigations and research. It has been demonstrated that the aqueous portion of *B. serrata* extract has hypolipidemic potential by lowering the level of total cholesterol in animal experiments. The serum cholesterol and triglyceride levels of rats were kept within a healthy range by *B. serrata* gum’s antihyperlipidemic activity (Pandey et al. 2005). According to research, NF-kB activity in atherosclerosis is inhibited by AKBA (Cuaz-Pérolin et al. 2008). In an in vitro investigation, AKBA is also known to have anti-adiposity properties since it causes lipolysis in mature human adipocytes. Peroxisome proliferator–activated receptor (PPAR)-γ2 expression was downregulated along with this event, and phenotypic markers were lost (Liu et al. 2013).

Further, the methanolic extract of *B. dalzielli* hutch stem bark possessed hypolipidemic activity via a significant decrease in triglycerides, total cholesterol, and low-density lipoprotein (LDL)-cholesterol levels in the treated rats, while the level of HDL increased significantly (Sani Jaafaru et al. 2018). Similarly, in clinical trials, *Olibanum* (from trees of the genus *Boswellia*) and *B. serrata* gum resins improved the profile picture of diabetic patients, by lowering total cholesterol, LDL, and triglycerides levels, while enhancing HDL levels, indicating that BA could be the potential substitute for hyperlipidemic agents with side effect (Azadmehr et al. 2014, Mehrzadi et al. 2016).

### Immunomodulatory activity

Immunomodulatory drugs include three main categories: immunosuppressants, immunoadjuvants, and immunostimulants. The immunosuppressants aim to restrain the immune response in patients suffering from autoimmune diseases or following organ transplantation to avoid rejection. The immunoadjuvants aim to strengthen the immune response by enhancing the duration or magnitude of a specific antigen as in the case of vaccines. As for immunostimulants, they help to enhance the immune response in patients suffering from immunodeficiency diseases or infections (Behl et al. 2021).

Formerly, investigations of the immunomodulatory activity of phytochemicals focused mainly on the potential anti-inflammatory effects in various inflammatory or autoimmune diseases. BAs were reported to have anti-inflammatory actions via inhibiting the 5-LOX (Siemoneit et al. 2009). This was a rationale for extending investigation on the potential immunomodulatory and anti-arthritic effects of BA, extracted from* Boswellia serrata* (*B. serrata*) gum resin, in carrageenan-induced paw edema and adjuvant-induced arthritis as an experimental model of rheumatoid arthritis in rats (Singh et al. 2008). Data from this study disclosed that BA managed to significantly reduce paw edema in both the carrageenan-induced and adjuvant-induced arthritis models in a dose-dependent fashion.

Other in vitro and in vivo studies ensued aiming to unravel the exact immunomodulatory role of BA and the implicated mechanism of action. In the study of Khajuria et al. (2008a, b), the oral administration of biopolymeric fraction (BOS 200) from *B. serrata* in mice led to immunostimulatory effects as revealed in the dose-dependent increase of the footpad thickness following the challenge with the sheep red blood cell antigen to induce delayed hypersensitivity reaction. An increase in the immunoglobulins IgG and IgM antibody titer was also observed, which was maximum with the high dose of BOS 200 (10 mg/kg). Incubation of macrophages with BOS 200 augmented the phagocytic function as revealed in the significantly higher phagocytic index. Lymphocyte immunophenotyping in the spleen depicted a significant increase in CD4 and CD8 following the administration of BOS 200. Also, the oral administration of BOS 200 at 3 and 10 mg/kg led to significantly higher levels of TNF-α, IFN-γ, and IL-4 in the serum.

In agreement with the aforementioned study, Gupta et al. (2011) revealed an immunoadjuvant effect for *B. serrata* BOS 2000 by enhancing the immune response to the weak antigen ovalbumin (OVA) in mice. Following 2 weeks of immunization, humoral response to BOS 2000 addition revealed significantly increased serum levels of OVA-specific IgG, IgG1, and IgG2a antibodies when compared with the OVA control group. The ex vivo splenocyte proliferation assay revealed a significantly heightened cell-mediated immune response as shown in the increased splenocyte proliferation in the OVA/BOS 2000-immunized mice, relative to the OVA control group. In a dose-dependent fashion, BOS 2000 showed a significant increase in the expression of CD80 and CD86 on the splenic macrophages. Lymphocyte immunophenotyping in the spleen illustrated significantly higher levels of CD4^+^ and CD8^+^ T cells upon BOS 2000 administration at the dose of 80 μg, when compared to their levels in the OVA-treated mice. Moreover, immunization of mice with BOS 2000 in addition to OVA led to a significant increase in the Th1 (IL-2 and IFN-γ) as well as Th2 (IL-4, IL-6, and IL-10) cytokines levels in the spleen cell culture supernatant, when compared to OVA immunization. Therefore, BOS 2000 can be regarded as a powerful immunoadjuvant to OVA in mice via its observed ability to enhance humoral and cell-mediated immune responses, which can offer a potential adjuvant in vaccines to combat infections caused by viruses, bacteria, or protozoa.

Another study carried out by Beghelli et al. (2017) investigated the potential immunomodulatory activity of seven standardized *B. serrata* gum resin extracts (containing 65% BA). Results of this study indicated that the* Boswellia* extracts did not induce B cell or lymphocyte proliferation in the absence of an activator when assessed via the in vitro carboxyfluorescein diacetate succinimidyl ester assay. However, the addition of *Boswellia* extracts to pokeweed mitogen–activated lymphocytes revealed an immunoadjuvant effect as shown in the significantly greater lymphocyte response. Moreover, the addition of one of the *Boswellia* extracts, in the absence of an activator, led to a significant increase of the FOXP3^+^ regulatory T (Treg) cells, which are proteins implicated in immune system response, indicating immunostimulant potential for BA.

The paradoxical immunomodulatory effects of *B. serrata* extract, acting as immunostimulant or suppressant, might depend on the extract concentration utilized where it was previously proposed that BA at low concentration tend to stimulate an immune response, as those utilized in the study of Beghelli et al. (2017) however, utilizing higher concentration suppressed the immune response (Ammon 2010).

In 2019, the outbreak of Coronavirus disease 2019 (COVID-19), caused by the novel SARS-CoV-2, was soon regarded as a global pandemic in 2020. Immunomodulators gained special attention in COVID-19 research due to the observed involvement of severe immune responses in some patients. It was noticed in certain severe cases of COVID-19 patients that a vast release of cytokines and chemokines occurs, a condition also known as cytokine storm that was regarded as the main cause of death in severe cases of COVID-19. This cytokine storm provokes an uncontrolled systemic inflammation in which the immune system aggressively attacks the body inducing acute respiratory distress syndrome, potentiating multiple organ failure, and eventually causing death (Xu et al. 2020). Though* B. serrata* is now being regarded as a potential immunomodulatory herb, which represents a promising therapeutic option for the treatment of COVID-19-associated complications; however, few studies proposed a probable therapeutic benefit for *B. serrata* in COVID-19 based on previously reported immunomodulatory actions in other diseases (Brendler et al. 2021, Gomaa et al. 2021). Yet, sufficient clinical data of actual investigation for BA benefits in COVID-19 patients is lacking.

So far and to the best of the authors’ knowledge, one double-blind randomized controlled trial (RCT) investigated the immunomodulatory effect of *B. serrata* extract as an adjunctive therapy to standard treatment in hospitalized patients with moderate COVID-19 (Barzin Tond et al. 2022). The treatment with *Boswellia* extract syrup (Inflawell®; standardized to include 40% BA) for 14 days successfully alleviated some of the COVID-19 clinical symptoms and showed a significant increase in oxygen saturation, as compared to patients who received a placebo. Noteworthy, the average duration of hospitalization, which is the primary outcome of the study, was significantly reduced in BA-treated patients, in comparison to the placebo group. The secondary outcome was to observe the effect of BA on the levels of inflammatory cytokines and PCR results by the end of treatment. It was recently reported that a low count of lymphocytes (lymphopenia) is probably linked to the severity of COVID-19 symptoms (Ghizlane et al. 2021). Barzin Tond et al. (2022) indicated the ability of BA treatment to significantly increase the lymphocyte count, and inversely, hamper the neutrophil-to-lymphocyte ratio, where this ratio is regarded as a beneficial prognostic factor in the early screening of severe illness in COVID-19 patients (Ciccullo et al. 2020, Kong et al. 2020). In adjunct, the levels of C-reactive protein, lactate dehydrogenase (LDH), IL-6, and TNF-α were significantly reduced in response to BA treatment, results which make it worthy to investigate the effect of BA in the case of the cytokine storm. The overall data on the BA effect in moderate COVID-19 cases is tempting and requires further investigations.

To conclude, the impact of *B. serrata* extracts or BA in COVID-19 patients offers a challenging area for investigators. Extending research to larger clinical trials covering both mild and severe cases of COVID-19 is needed to confirm the currently available findings. Moreover, using different doses of BA will help to identify the nature of its potential immunomodulatory actions, being stimulant or suppressant, and the probable usefulness in a cytokine storm.

### Antidiabetic effects

Many preclinical studies have revealed promising hypoglycemic effects for different forms of extracts from the gum resin of* B. serrata* or related species against type 1 (T1DM) and type 2 diabetes mellitus (T2DM) experimental models, either administered solely or as an add-on to known antidiabetic agents.

In the study of Samala and Veeresham (2016), a pharmacokinetic study revealed that the co-administration of BA with glyburide increased the *C*_max_ and *t*_*1/2*_ of glyburide, in addition to reducing its clearance. Since glyburide is a cytochrome P450 (CYP) 3A4 substrate, and BA is an inhibitor of this liver microsomal enzyme (Frank and Unger 2006, Zhou et al. 2010). Thus, BA can reduce glyburide metabolism, and increase its bioavailability. A pharmacodynamic study also revealed that the combination of BA with glyburide enhanced the glucose-lowering ability of glyburide in diabetic rats.

As a single agent administered to diabetic rats, Kherouf et al. (2021) demonstrated that *B. serrata* gum resin powder managed to significantly lower serum glucose levels in rats subjected to a single high dose of streptozotocin (STZ). Furthermore, the serum insulin levels were significantly higher in the* B. serrata*–treated diabetic rats, indicating the protective impact of *B. serrata* against STZ-induced destruction of pancreatic β cells.

Inflammation and immune system players are involved in the pathogenesis of T1DM and T2DM. Hence, it was appealing to investigate the potential benefit of *Boswellia* extracts owing to their previously evident anti-inflammatory and immunomodulatory effects in different ailments. Consistent with this notion, many studies have revealed that when BA was given solely, they managed to achieve significant results via interrupting the chronic inflammation implicated in DM, thus attenuating its progression, and reducing blood glucose levels. In the study of Shehata et al. (2011), which adopted the multiple low-dose STZ, experimental models of T1DM, administration of *B. serrata* alcoholic extract for 10 days, concurrently with multiple low-dose STZ, showed significantly lower serum glucose levels and almost normal pancreatic islets, when compared to mice treated with STZ only. The authors attributed the reported effects to the ability of* Boswellia* extract to inhibit the colony-stimulating factors; granulocyte colony–stimulating factor, which affects the production of the master transcription factor, NF-κB, in addition to abrogating neutrophil infiltration and apoptosis of the β cells. In a later study adopting the same experimental design, Shehata et al. (2015) demonstrated that two BA out of four investigated did not significantly alter the blood glucose level. These two BAs were β-BA and acetyl-β-BA, which do not have the 11-keto group in their molecule. However, the other two BA having the 11-keto group; 11-keto-β-BA and β-AKBA, managed to significantly reduce the blood glucose levels in STZ-treated mice. The authors credited the anti-diabetic potential for β-KBA and β-AKBA to their inhibitory effect on STZ-induced increase in inflammatory mediators, hence, halted insulitis and hyperglycemia. To further investigate the anti-diabetic mechanism of β-KBA, Shehata et al. (2017) adopted a genetically engineered model of autoimmune T1DM, known as non-obese diabetic mice, which is close to the human T1DM. In this study, β-KBA managed to curtail CD3 lymphocyte infiltration and insulitis, thus, protecting the pancreatic β cells against destruction. These results can provide a promising initiative for T1DM patients.

In an experimental model of T2DM, Gomaa et al. (2019) elaborated that* B. serrata* extracts managed to significantly lower the serum glucose and insulin levels as well as reduce the homeostatic model assessment of insulin resistance only at the doses of 300 and 400 mg/kg and not 200 mg/kg. In the later study done by Khan et al. (2022), a more comprehensive evaluation was done for the antioxidant, anti-hyperlipidemic, and anti-diabetic effects of specifically β-BA and β-KBA, extracted from *Boswellia sacra* (*B. sacra*), in a high-fat diet and low-dose STZ T2DM model. Both β-BA and β-KBA effectively reduced the serum glucose levels in the diabetic rats without inducing hypoglycemia, in addition to reducing the lipid profile indices except for HDL-C which was increased dose-dependently. From the perspective that oxidative stress is a key player involved in the incidence of impaired glucose tolerance, metabolism, and insulin resistance, Khan et al. (2022) elaborated that the hypoglycemic activity of β-BA and β-KBA might be linked to the attenuation of serum malondialdehyde (MDA) and enhancing SOD levels, following their administration to the diabetic rats. To unravel their molecular mechanism, both in silico and in vitro studies revealed that the anti-diabetic effects of β-BA and β-KBA may be mediated via inhibiting the dipeptidyl peptidase 4 enzyme, thus, increasing the levels of the incretin hormone glucagon-like peptide 1.

Clinical studies on the anti-diabetic effects of BA are far fewer than the pre-clinical ones. Schrott et al. (2014) published the first case report on the effect of *B. serrata* extract, which contained β-BA and β-KBA, in a 50-year-old female patient diagnosed with late-onset autoimmune diabetes of adults (LADA). The addition of this extract to her daily insulin therapy for 8.5 weeks led to a significant decline in the biomarker of LADA, tyrosine phosphatase antibody (IA_2_-A), back to normal levels, in addition to the decline in blood glucose levels and HbA_1C_, when compared to insulin therapy alone. Another case report published by Franic et al. (2020) investigated the effect of *B. serrata* extract on a male patient diagnosed with LADA and having positive glutamic acid decarboxylase (GAD65) antibodies, another biomarker for LADA. After 9 months, this patient had lower fasting blood glucose levels and HbA_1C_ and reduced GAD65 by about 25%. These two case reports confirm the ability of *B. serrata* extract to lower the markers of insulitis, IA_2_-A, and GAD65, in patients with LADA, thus, improving the disease outcomes. However, larger clinical studies on patients with LADA are needed to fortify the published case reports.

An RCT performed by Azadmehr et al. (2014) investigated the effect of Olibanum gum resin, from *B. serrata*, in T2DM patients. Following 12 weeks, the trial results indicated that Olibanum gum resin capsules managed to lower the fasting glucose levels, HbA_1C_, and insulin in the intervention group when compared with the placebo one. In the same context, a later RCT investigated the anti-diabetic effect of *B. serrata* standardized extract (containing 60% BA) in T2DM patients (Mehrzadi et al. 2018). Opposite results were shown in this trial where the complementary administration of the 60% BA capsules at the dose of 250 mg twice daily for 8 weeks did not show a significant difference from the placebo regarding the fasting blood glucose, insulin, and HbA_1C_ levels. The authors attributed these results to the possibility of the small sample size implicated in the study, especially since there was an observed reduction in the fasting blood glucose and HbA_1C_ levels in diabetic patients, though non-significant, compared to the placebo group.

Although the currently available studies raise expectations toward the potential use of BA or any of its pharmacologically active ingredients to halt the progression of autoimmune reaction in T1DM or insulin resistance in T2DM, thus interrupting the occurrence of overt diabetes or aid in the reduction of hyperglycemia; however, the advance to a large double-blind RCT is still lacking as well as studies on a larger number of patients with LADA, which account for about 9–12% of all diabetes patients.

### Hepatoprotective effects

Preclinical studies demonstrated hepatoprotective impact for BA against different models of hepatotoxicity via tackling oxidative stress, and inflammatory and apoptotic indices. Carbon tetrachloride is a known toxin used to induce hepatotoxicity in animals. Eltahir et al. (2020) investigated the beneficial effects of *Boswellia serrata* gum resin against carbon tetrachloride–induced hepatic injury in rats. It was revealed that the gum resin managed to reverse the elevation in the serum levels of hepatocyte integrity markers; alanine aminotransferase (ALT), aspartate aminotransferase (AST), and LDH. Hepatoprotection by the gum resin was mediated via enhancing the hepatic antioxidant capacity and catalase activity, together with reducing lipid peroxidation and the expression of hepatic transforming growth factor beta (TGF-β), NFκB, TNF-α, and IL-6, thus curbing hepatic oxidative stress and inflammation.

The high-fat diet (HFD) is an experimental model of non-alcoholic fatty liver disease (NAFLD) that mimics steatohepatitis occurring in humans. Fatty liver diseases involve fat accumulation in the liver as well as excessive free radicals formation and inflammatory cell infiltration. The protective impact of a standardized extract of *B. serrata* including 65% BA was investigated in an HFD-induced NAFLD rat model (Zaitone et al. 2015). Eight weeks of administration of BA resulted in ameliorated levels of serum ALT and AST, which reflects lower hepatocellular damage, thus reducing leakage of liver function enzymes to serum. Detailed investigation of the protective mechanism of BA revealed dose-dependent anti-inflammatory and antioxidant potential for BA, where increased hepatic reduced glutathione (GSH) levels, concomitantly with reduced serum levels of the inflammatory mediators: TNF-α, IL-6, and COX-2 and hepatic MDA levels was observed in the groups treated with BA in addition to lower immunoreactivity to 4-hydroxy-2-nonenal (4-HNE) and iNOS in hepatic tissues, compared to the HFD-treated rats.

Another study utilizing the standardized extract of *B. serrata* including 65% BA successfully abrogated doxorubicin-induced hepatotoxicity in mice (Barakat et al. 2018). Doxorubicin, like all chemotherapeutic agents, can be toxic to normal cells via enhancing free radicals’ formation. The liver is one of the organs that are susceptible to doxorubicin toxicity as it is significantly metabolized through the liver. Barakat et al. (2018) revealed that BA exerted a dose-dependent hepatoprotective effect, mediated through reducing lipid peroxidation as evidenced by the declined MDA levels, concurrent with enhancing the expression of nuclear factor E2–related factor 2 (Nrf2). Increased Nrf2 expression can advocate for the subsequent expression of genes encoding for antioxidant enzymes such as heme oxygenase 1 (HO-1). The antioxidant effect of BA resulted in curbed caspase-3 activity and hepatocellular death, as well as declined serum levels of the liver function enzymes.

Acetaminophen is a known causative agent of hepatotoxicity when ingested in overdose owing to the production of large amounts of n-acetyl-p-benzoquinoneimine which depletes the natural antioxidant defense molecules. Chen et al. (2016) revealed the hepatoprotective effect of BA administered in the diet to mice against acetaminophen-induced hepatotoxicity. This was evident via reduced serum AST, ALT, and C-reactive protein and attenuated oxidative stress and inflammatory markers in liver tissues. Chen et al. (2016) tracked NFκB/JNK/TLR signaling pathways involved in acetaminophen toxicity, and results indicated the ability of BA to downregulate the expression of NFκB p65 and p-JNK, whereas BA high dose only managed to curtail the expression of TLR3, TLR4, and myeloid differentiation primary response 88 (MyD88).

Kumar et al. (2017) investigated the hepatoprotective effect of β-AKBA in a benzo(a)pyrene-induced hepatic dysfunction rat model since benzo(a) pyrene is a known harmful hydrocarbon present in engine smoke, cigarette smoke, and others to which humans can be subjected daily affecting their livers. Administration of β-AKBA managed to significantly alleviate the elevation in liver function enzymes and improve the histoarchitectural damage induced by benzo(a) pyrene in liver tissues, yet it was not capable of alleviating oxidative stress indices. In the same context, Thabet et al. (2022) investigated the possible hepatoprotective effect of BA tablets (*B. serrata* extract including 65% BA) against two environmental pollutants; bisphenol-A and gamma-radiation to which human beings are exposed in their daily life. These two pollutants are known to induce tissue and organ damage via the induction of oxidative stress and inflammation. In addition to diminishing serum AST and ALT, BA managed to significantly reduce the hepatic MDA, IL-6, and TNF-α levels, which correlated with replenished GSH levels. To elaborate on how BA exerted its hepatoprotective actions and reduced hepatic steatosis, mechanistic investigations revealed upregulation of the hepatic PPAR-α/p38 signaling axis following BA treatment. p38 is a member of the MAPK family which was previously reported to ameliorate hepatic steatosis in pediatric patients via antagonizing JNK and upregulating PPAR-α (Xiao et al. 2017). Additionally, PPAR-α is known to activate β-oxidation of free fatty acid and reduce the expression of genes involved in lipogenesis, thus, responsible for lowering serum lipids and fat accumulation in the liver. Therefore, upregulating PPAR-α and p38 expression can explain how BA counteracted hepatic steatosis.

Ischemia-reperfusion has detrimental outcomes on multiple organs including the liver. In the study performed by Monir et al. (2022) renal ischemia reperfusion-induced hepatic injury was revealed through the elevated serum liver function enzyme levels as well as oxidative stress indices and inflammatory cytokines levels. Administration of BA standardized extract managed to reverse the ischemia reperfusion-induced damage as a witness in the reduction of the aforementioned markers. Increased expression of TLR9 was stated following renal ischemia-reperfusion, which can be attributed to the increased release of mitochondrial DNA from the apoptotic cells. This is ensured by the activation of inflammatory response and promoting liver injury (Bakker et al. 2015). Herein, Monir et al. (2022) reported the reduced expression of TLR9 in response to BA treatment, which can advocate for its hepatoprotective mechanism.

Recently, BAs showed promising hepatoprotective effects in a mouse model of experimentally-induced alcoholic liver disease. BAs improved lipid profile and hepatic inflammation, ROS and apoptosis, through decreasing CYP2E1, nicotine adenine dinucleotide phosphate oxidase (NOX) 1/2/4, p38 MAPK, sterol regulatory element-binding protein-1c levels, and the expression of miR-155, while increasing PPARα levels (Salama et al. 2023).

Regarding the possible hepatoprotective impact of BA in patients, an RCT was performed on T2DM patients supplemented with *B. serrata* gum resin for 6 weeks and revealed a significant decline in serum glutamic pyruvic transaminase and serum glutamic-oxaloacetic transaminase levels, illustrating a positive hepatoprotective effect for *B. serrata* (Ahangarpour et al. 2014). However, limited clinical data is available regarding the impact of BA in patients with hepatic dysfunction.

To conclude, *Boswellia* gum resin or BA treatment was shown to be hepatoprotective in various experimental models of hepatic injury which are close to the clinical picture of liver diseases in humans. However, the advance from pre-clinical to clinical investigations is still insufficient.

### Anti-asthmatic activity

Pathophysiology of asthma involves the differentiation of Th2 cells, ensued by the production of IL-4, IL-5, and IL-13 in the bronchi (Gour and Wills-Karp 2015, Pelaia et al. 2019). These inflammatory cytokines enhance the recruitment of inflammatory cells into the lung, which leads to airway inflammation, narrowing of the lumen, increased mucus secretion, and hyper-responsiveness. BA seems to be an appealing pharmacological option owing to its established anti-inflammatory actions and previously reported inhibition of LOX-5 (Siemoneit et al. 2009), which is a known target of the leukotriene modifiers anti-asthma class. Liu et al. (2015) investigated the anti-asthmatic activity and molecular mechanism of BA in an experimental model of asthma in mice. Results of this study confirmed the ability of BA, in a dose-dependent manner, to attenuate hyper-responsiveness, inflammatory cell infiltration in the lung tissues and bronchoalveolar lavage fluid (BALF), as well as the serum levels of IgE, IL-4, IL-5, and IL-13. Phosphorylation of STAT-6 allows its translocation to the nucleus and subsequent induction of GATA binding protein 3 (GATA3) gene transcription. The GATA3 is known to be a master regulator of Th2 cell differentiation, thus, enhancing GATA3 expression can lead to increased production of the inflammatory cytokines IL-4, IL-5, and IL-13 (Barnes 2011). This was evident in the experimental asthma mice model in both studies of Liu et al. (2015) and Zhou et al. (2010), in which BA managed to halt the GATA3/STAT6 axis, thus, introducing a possible explanation for its potent anti-asthmatic effect.

Since β-AKBA was reported to be the most potent BA among the six different types (Iram et al. 2017), it was noteworthy to investigate its effect on asthma. Recently, the study of Suther et al. (2022) investigated the potential anti-asthmatic effect of β-AKBA in an allergic asthma model in BALB/CJ mice. The administration of β-AKBA managed to reduce mucus production and alleviate lung tissue inflammation and bronchial smooth muscle remodeling. In addition, treatment with β-AKBA managed to reduce hyper-responsiveness and bronchoconstriction as revealed through the increased resistance to methacholine bronchoprovocation test. In-depth investigations revealed the ability of β-AKBA to reduce leukocytes and eosinophils count as well as IL-4 and IL-5 levels in the BALF. The authors anticipated that the anti-asthmatic effects of β-AKBA, at least partially, might involve modulating the gut microbiome, via increasing *Bifidobacterium pseudolongum*, thus, attenuating airway inflammation.

Scarce data is available regarding the effect of *Boswellia* gum resin or its extracts in asthmatic patients. The most recent double-blind RCT investigated the effect of *B. serrata* gum resin extract (30% β-AKBA) in asthmatic patients, yet, in combination with *Aegle marmelos* fruit extract in a 1:1 ratio; a formula known as AlvioLife® or LI13109F (Yugandhar et al. 2018). Notably, this herbal formulation was tested in vitro and in vivo before clinical investigation. Results of this comprehensive study revealed the ability of the 1:1 mixture of *B. serrata* gum resin extract and *A. marmelos* fruit extract to achieve the most potent inhibitory action on 5-LOX, showing better synergistic activity in comparison to other blending ratios. This was confirmed by the ability of LI13109F to significantly attenuate the over-expression of 5-LOX in the lipopolysaccharide-induced THP-1 human monocyte cell line. In parallel, the pre-clinical study illustrated the ability of LI13109F to curb the Sephadex® LH-20-induced bronchial inflammation in rats, as evidenced in the abrogated levels of IL-4 and enhanced levels of IFN-γ in lung tissues, as well as the significant dose-dependent drop in granulocytes count in BALF. The clinical study adopted the improvement in the score of the asthma quality of life questionnaire (AQLQ) as the primary endpoint. Successfully, the supplementation of LI13109F to asthmatic patients for 56 days led to significant improvement in the AQLQ scores, as compared to the placebo. For the secondary endpoints, the LI13109F significantly improved the peak expiratory flow rate and the forced expiratory volume in 1 second. Moreover, the supplementation of LI13109F led to significantly increased serum levels of IFN-γ concomitantly with reduced levels of IL-4, when compared with the placebo. Herein, BA extracted from *B. serrata* gum resin illustrated therapeutic potential in asthma through different studies, yet larger clinical trials need to be implemented.

### Clastogenic activity

Clastogenic activity is a form of mutagenesis that refers to the ability of a drug or chemical to induce chromosomal damage. This can lead to the insertion, deletion, or re-arrangement of chromosomes, events that can end up in cancer. Literature review denotes the availability of few reports on* B. serrata* gum resin extracts regarding its clastogenic or anti-clastogenic activity. However, Alluri et al. (2019) illustrated in their study the impact of a novel combination of the acidic and non-acidic fractions of *B. serrata* gum resin, known as serratrin (LI13019F1) for its mutagenic and clastogenic activity. The genotoxicity impact of three oral doses of LI13019F1 was assessed via the erythrocyte micronucleus assay in the mouse bone marrow which examines the presence of micronuclei. These micronuclei may comprise chromosome fragments resulting from DNA breakage (clastogens) or intact chromosomes resulting from the dismantling of the mitotic apparatus (aneugens). In this context, smears of bone marrow samples revealed that the oral doses of LI13019F1 to 2000 mg/kg did not change the number of immature polychromatic erythrocytes in both male and female mice. In addition, the percentage of micronucleated polychromatic erythrocytes did not show a significant difference from the control group, yet it was significantly lower when compared to cyclophosphamide-treated mice. Thus, these observations add to the safety profile of *B. serrata* extracts, owing to the absence of genotoxic potential manifested in the absence of clastogenic or DNA damage capacity.

Recently, and in alignment with previous studies to confirm the safety of *Boswellia* extracts, the work of Dodda et al. (2021) investigated the safety of a water-soluble extract of* B. serrata* gum resin, known as LI51202F1, through several toxicity studies. The micronucleus assay in mouse bone marrow erythrocytes revealed that the resin extract is neither mutagenic nor clastogenic, assuring its broad-spectrum safety.

In a matter of fact, the currently available data denote that BA has anti-clastogenic activity rather than a clastogenic effect. As per the study of Ganguly et al. (2011), BA at the dose of 200 mg/kg not only managed to suppress the cyclophosphamide-induced genotoxicity in mice, but it did not confer a genotoxic effect too. This was shown through the ability of BA treatment to significantly halt the formation of micronuclei by the clastogen, decrease chromosomal break and ring formation, as well as alleviate chromosomal aberrations in cyclophosphamide-treated mice. In addition, BA alone in the absence of cyclophosphamide neither enhanced micronucleus formation nor conferred chromosomal aberration.

Herein, the absence of clastogenic activity for *B. serrata* gum resin extracts in the pre-clinical studies and inversely, the anti-clastogenic potential for BA can pave the way to stepping forward and investigating the potential therapeutic benefits of BA safely in patients, without the fear to cause any DNA aberrations.

### Useful actions on skin and psoriasis

BAs exhibit anti-inflammatory activity in several inflammatory conditions, including rheumatoid arthritis, osteoarthritis, and asthma. Active substances are currently administered to psoriatic and eczematous patients topically. Currently, a topical administration of BAs is used in psoriatic and eczematous patients (Togni et al. 2014). Psoriasis is a non-communicable autoimmune that manifests as a chronic, recurrent, inflammatory skin disease (Schön and Boehncke 2005). Sharply defined erythematous plaques covered in silvery or opalescent scales are the hallmark skin lesions of psoriatic patients (Schön et al. 2005). The infiltration of polymorphic neutrophils in the skin supports the inflammatory response. The chemokines and lymphokines generated by keratinocytes and T lymphocytes, respectively, cause neutrophils to activate, which in turn causes lymphocytes and keratinocytes to activate. This vicious cycle is only present in acute lesions but is nonetheless connected to ongoing inflammation (Schön and Ludwig 2005). Immunosuppressive medications such as methotrexate, cyclosporine, and fumaric acid esters are used to treat psoriasis (Belge et al. 2014). The molecular understanding of signaling has improved recently; nevertheless, pathways implicated in the psoriasis etiology have led to the investigation of biological treatments. These include immune suppressive drugs (alefacept) and anti-cytokine treatments TNF therapies (adalimumab, etanercept, infliximab, and ustekinumab) (Papoutsaki and Costanzo 2013). Alternatively, to treat psoriatic and eczematous symptoms, anti-inflammatory molecules can be applied topically. By directly addressing inflammatory processes and producing a noticeable calming reaction, this method is a viable alternative to systemic treatments (Samarasekera et al. 2013). Only 5% of patients acquire biological treatments, which account for 67% of the cost of all psoriasis drugs, according to a recent study. In contrast, topical drugs are most frequently used and are responsible for 18% of total expenditures. Additionally, because approximately 10% of patients need to get at least three topical medication changes over a year to achieve medical benefits, developing and synthesizing novel anti-inflammatory agents for topical use is both a medical necessity for the treatment of psoriasis and a potential cost-saving approach (Mustonen et al. 2013). *Boswellia serrata* gum resin extracts (BSEs) are used in traditional ayurvedic medicine to treat inflammatory diseases. BAs are pentacyclic triterpenes that majorly contribute to the *Boswellia* resin composition. There were 12 discovered types of BAs present in *Boswellia*: α-BA, β-BA, acetyl-α-BA (AαBA), acetyl-β-BA (AβBA), lupeolic acid, acetyl-lupeolic acid, acetyl-9-11-dehydro-α-BA, 11-dehydro-α-BA, acetyl-9-11-dehydro-β-BA, 9,11-dehydro-β-BA, 11-keto-β-BA (KBA), and acetyl-11-keto-β-BA (AKBA). Amongst these BAs, KBA and AKBA are the most active (Büchele and Simmet 2003).The pentacyclic triterpenes BAs, of which various forms have been described by analytical techniques, including α- and β- configured BAs, are the predominant component of the lipophilic fraction of olibanum (Poeckel and Werz 2006). The efficacy of BSEs for the treatment of a wide range of inflammatory disorders, such as inflammatory bowel disease, rheumatoid arthritis, osteoarthritis, and asthma, is supported by animal research and preliminary clinical trials (Ammon 2010). Additionally, *Boswellia* resin has been proven to possess anti-inflammatory properties that prompt the alleviation of symptoms of ulcerative colitis, Crohn’s disease, Parkinson’s disease, Alzheimer’s disease, etc. making it an essential ingredient employed in alternative and complementary medicine approaches (Siddiqui 2011), The main anti-inflammatory effects of BAs are thought to be caused by KBA and AKBA’s inhibition of 5-LOX, which suppresses the production of leukotrienes (Abdel-Tawab et al. 2011). There is some evidence of an indirect BA action leading to an irreversible Ca2+-mediated inhibition of 5-LOX, albeit the molecular mechanism needs to be completely clarified (Altmann et al. 2004). Inhibiting human leukocyte elastase, which is produced in inflammatory and hypersensitive environments, seems to be another way that BAs support their antiphlogistic properties (Ammon 2006) Through its direct inhibition of IKK in activated human monocytes, AKBA transmits suppression of NF-kB and subsequent downregulation of TNF-α production (Syrovets et al. 2005a). It is noteworthy that the suppressive effects on NF-kB signaling have been validated when the IKK inhibition by BAs has been studied in the CD18 hypomorphic (CD18[hypo]) mouse model of psoriasis, suggesting that targeting NF-kB with BAs may be an effective strategy (Wang et al. 2009).

### Activity in Crohn’s disease

Inflammatory bowel disease (IBD) is a long-term gastrointestinal inflammatory disorder that experiences remissions and relapses in a cycle. The two most prevalent forms of IBD are ulcerative colitis and Crohn’s disease, both of which are characterized by increased, uncontrolled intestinal inflammation that worsens patients’ quality of life and necessitates prolonged medication and/or surgical procedures. Although Crohn’s disease–related inflammation can affect any part of the gastrointestinal tract, from mouth to the anus, it most frequently affects the distal small bowel and/or colon. The mucosa, submucosa, and muscularis propia of inflamed bowel samples taken from patients with active Crohn’s disease exhibit transmural inflammation and a significant aggregation of acute and chronic inflammatory cells (Baumgart and Carding 2007, Vucelic 2009). To apprehend this mechanism in which BAs exhibit their anti-inflammatory properties, it is vital to scrutinize the pathogenesis of Crohn’s disease. Although there is overwhelming agreement that IBD is caused by a complex interaction of four main factors, including multiple genetic variations, changes in the composition of the intestinal microbiota, changes in the environment, and over-reactivity of the intestinal mucosal immune response, the etiology of IBD is, unfortunately, not fully understood (Strober and Fuss 2011, Haag and Siegmund 2014). Due to changed intestinal microbiota, genetically predisposed patients develop an excessive and uncontrolled immune response in the gastrointestinal tract, which results in chronic intestinal inflammation. Similar to other inflammatory disorders, the wide variety of inflammatory mediators, such as cytokines, chemokines, leukotrienes, and PGs, as well as reactive oxygen and nitrogen species, are responsible for the pathophysiology of IBD (Xavier and Podolsky 2007). Crohn’s disease is characterized by a tenacious immune response against antigens of luminal bacteria (Baumgart 2017), (Wallace et al. 2014). Predominantly, T cells become hyperactive and excessively release inflammatory cytokines such as interleukin (IL)-12 and IFN-γ which prompt a T helper type 1 cell response. TNF-α is also released and has been associated with an elevation in CD4+ and FoxP3+ regulatory T cells in the mucosa of Crohn’s disease patients (de Souza and Fiocchi 2015). Moreover, IL34 has been directly affiliated with Crohn’s disease by inducing TNF-α AND IL6 expression. Amongst all the Crohn’s disease pathogenesis-correlated ILs, IL-12, and IL-23 are potential targets for therapy (Fina et al. 2011), (Marafini et al. 2015, Neurath 2019). Pharmacological studies have established that boswellic extracts are beneficial in chronic inflammatory disorders such as Crohn’s disease (Ammon 2010). According to Roy et al., a standardized boswellic extract posed a successful anti-inflammatory effect on 133 normally TNF-α-induced genes in human microvascular endothelial cells. Normally, TNF-α would acutely induce 522 genes and repress 141; however, 133 of these genes that contribute to inflammation were observably sensitive to boswellic extract application. Moreover, boswellic extract has proved to inhibit TNF-α-induced expression of apoptotic mediators and metalloproteinases whose function concerns proteolysis (Roy et al. 2005). Furthermore, according to Chevrier et al., BAs solubilized in sesame oil instigated inhibition of IL-2 and IFN-γ along with IL-4 and IL-10 potentiation, as opposed to the cellular toxicity marked when ethanol was used as the solvent. In this context, IL-4 impedes macrophage activation while IL-10 diminishes the development of T helper type 1 cells (Chevrier et al. 2005). Another study revealed that a crude methanolic boswellic resin suppressed TNF-α, IL-1, and IL-6 in peripheral blood mononuclear cells and had downregulated IL-12 and IFN-γ (Gayathri et al. 2007). In addition, Khajuria et al. proved that boswellic extract components subside the immune system activity through suppression of activation, development, and differentiation of T cells responsible for the production of IL-1, IL-2, IL-4, and IL-6 (Khajuria et al. 2008a, b). Furthermore, humoral immunity plays a vital role in the pathogenesis of Crohn’s disease in which CD4 T cells promote plasma cell differentiation in an IL-2-dependent manner. Then, IL-21 prompts B cells to express granzyme-B, a factor contributing to the further cytotoxic progression of intestinal epithelial damage seen in Crohn’s disease (Cupi et al. 2014). By testing humoral antibody production in mice serum after treatment with sheep erythrocytes, Sharma et al. deduced that a single oral dosage of 50 to 200 mg/kg of a BAs mixture on the sensitization day engendered a reduction in hemagglutinating antibody titers on the fourth day. Higher doses of 100 to 200 mg/kg produced even greater reductions in antibody production. In addition, oral doses of 25, 50, and 100 mg/kg of a mixture of BAs were administered for 5 days around the time of immunization. At 100 mg/kg, there was a remarkable reduction in primary and secondary complement-fixing antibody titers (Sharma et al. 1996). Moreover, the primary active derivatives have been identified as KBA and AKBA (Abdel-Tawab et al. 2011), and many modes of action have been shown: inhibition of 5-LOX, immune system effects such as decreased levels of cytokines (IL and TNF-α), lessened complement system and leukocyte elastase activities, decreased ROS production and P-selectin-mediated recruitment of inflammatory cells (Ammon 2010).

### Activity in Alzheimer’s disease

Emerging research studies have investigated the potential therapeutic correlation between BAs in *Boswellia* resin and a variety of neurodegenerative diseases, including Alzheimer’s disease. To begin with, it is vital for building comprehension of the pathophysiology of Alzheimer’s disease. Alzheimer’s disease is predominantly characterized by soluble amyloid-β (Aβ) senile plaque formation which occurs upon the development and accumulation of neurofibrillary tangles (NFTs) in the brain (Association As 2019). As a result, synaptic and mitochondrial functions become impaired and defective, and the levels of ROS increase, aggravating intracellular oxidative stress. In Alzheimer’s brains, the accumulation of tau and Aβ proteins is a predominant hallmark (Boutajangout et al. 2011, Huang and Mucke 2012). Although tau is a naturally beneficial protein that stabilizes microtubules, preserves DNA, and promotes overall neuronal health (Avila 2016, Zhou and Wang 2016) upon the induction of inflammatory status, the tau protein is cleaved into fragments that may pile up in the brain and engender neurodegeneration. In response to certain stimuli, astrocytes and microglial cells recruit inflammatory mediators such as TNF-α, PGs, IL-6, and ROS which promote neurotoxicity. This could perturb the ubiquitin-proteosome pathway which results in protein aggregation accumulation that progresses to neuroinflammation and neuronal apoptosis, if not dispensed. Caspase proteolysis of tau also causes protein aggregates as mentioned previously (Metcalfe and Figueiredo-Pereira 2010). In addition, tau aggregation may be prompted by polyanions that provoke charge compensation of certain regions of the tau chain. In turn, hyperphosphorylated tau forms NFTs (Mandelkow and Mandelkow 2012). According to Karima et al., β-BA possesses restorative and preservative effects on neurons (Karima et al. 2010). It enhances the branching of neurites and boosts tubulin polymerization. Moreover, β-BA fortifies microtubule polymerization and promotes their lengthening, therefore preventing axonal degradation (Karima et al. 2012). Another rodent study revealed that AKBA downregulates the expression of beta-site APP cleaving enzyme 1 (BACE1) which is an enzyme responsible for cleaving amyloid-precursor protein (APP) hence preventing the accumulation of Aβ proteins in the brain. AKBA plays a vital role in the suppression of inflammatory mediator release granting it anti-inflammatory and antioxidant properties (Wei et al. 2020). Furthermore, utilizing primary fetal human cell lines with astrocytes exhibiting streptozotocin-induced Alzheimer’s features, α-BA demonstrated a significant reduction in tau hyperphosphorylation and ROS production, and enhanced cell division (Fathi et al. 2016). Moreover, the formation of senile plaques provokes the release of pro-inflammatory mediators, such as ILs, cytokines, and chemokines, which promote inflammation and exacerbate AD (Siddiqui et al. 2021). Alzheimer’s brains also express elevated levels of 5-LOX, specifically in the hippocampus and cortex regions (Ikonomovic et al. 2008), (Firuzi et al. 2007). The arachidonic acid pathway is another vital driver of inflammation. Upon stimulation by inflammatory mediators such as ILs, IFN-γ, and TNF-α, a membrane-associated enzyme known as phospholipase A2 catalyzes the liberation of arachidonic acid from cellular membranes which can be further oxidized by various oxygenase enzymes (Turman and Marnett 2010). COX-2 and 5-LOX are the two chief enzymes in the arachidonic acid oxidation pathway accountable for producing PGs and leukotrienes which in turn yield specific inflammatory symptoms (Ammon 2016). Leukotrienes deposition in the brain modifies brain tissue pathology (Di Gennaro et al. 2004, Michael et al. 2019). Multiple research papers tackled the therapeutic potential of BAs on Alzheimer’s through the biological targeting of 5-LOX and COX enzymes. According to Moritz Verhoff et al., BAs repress prostaglandin synthesis through the impediment of COX enzymes’ inflammatory activity (Verhoff et al. 2014). Another paper stated that AKBA suppressed oxidative stress-instigated neuronal injury and cognitive impairment due to its antioxidant and anti-inflammatory effects (Bishnoi et al. 2005, Sayed and El Sayed 2016). In addition, AKBA has substantially diminished the levels of inflammatory markers such as 5-LOX, TNF-, IL-6, and ameliorated cognition in lipopolysaccharide-induced neuroinflammation rodent models (Marefati et al. 2020).

## Conclusion

Multiple preclinical investigations and a wide range of clinical trials have shown that BAs, the pentacyclic triterpenic acids that include α-,β-,γ-BA,acetyl-β-BA, KBA, AKBA, and so on, have a wide range of pharmacological actions against many chronic diseases. They can attack multiple mechanisms that contribute to disease progression. Numerous chronic diseases owe a great deal to the actions of NF-B, MAPK, Erk-1/2, TNF-α, etc., all of which were found to be affected by BA treatment. Still, doubts about the compound’s pharmacokinetic qualities have had significant chilling effects on the road to developing it as an effective medication. Many studies have been launched to find ways through these obstacles, but progress is gradual, and there is a great deal of focus required.

## Data Availability

Not applicable

## Abbreviations

BA: Boswellic acid
AKBA: Acetyl-11-keto-β-BA
ROS: Reactive oxygen species
Bcl-2: B cell leukemia/lymphoma 2 protein
Bax: Bcl-2-associated X
PC: Pancreatic cancer
Akt: Ak strain transforming
ERK: Extracellular signal–regulated kinase
AR: Androgen receptor
PARP: Poly(ADP)-ribose polymerase
MAPK: Mitogen-activated protein kinase
LC: Lung cancer
JNK: c-JUN N-terminal kinase
STAT: Signal transducer and activator of transcription
TNF-α: Tumor necrosis factor-alpha
LOX: 5-lipoxygenase
iNOS: Inducible nitric oxide synthetase
SOD: Superoxide dismutase
PGs: Prostaglandins
IKK: Inhibitory κB kinase
LDL: Low density lipoprotein
BOS 200: Biopolymeric fraction
OVA: Ovalbumin
RCT: Randomized controlled trial
LDH: Lactate dehydrogenase
T1DM: T2DM type 1 and type 2 diabetes mellitus
CYP: Cytochrome P450
STZ: Streptozotocin
MDA: Malondialdehyde
LADA: Late-onset autoimmune diabetes of adults
GAD65: Glutamic acid decarboxylase
ALT: Alanine aminotransferase
AST: Aspartate aminotransferase
HFD: High-fat diet
NAFLD: Non-alcoholic fatty liver disease
GSH: Reduced glutathione
4-HNE: 4-hydroxy-2-nonenal
COX-2: Cyclooxygenase 2
Nrf2: Nuclear factor E2–related factor 2
HO-1: Heme oxygenase 1
BALF: Bronchoalveolar lavage fluid
GATA3: GATA-binding protein 3
IBD: Inflammatory bowel disease
NFTs: Neurofibrillary tangles
Aβ: Amyloid-β
BACE1: Beta-site APP cleaving enzyme 1
APP: Amyloid-precursor protein
